# Prevalence and correlates of burnout among collegiate cycle students in Sri Lanka: a school-based cross-sectional study

**DOI:** 10.1186/s13034-018-0238-z

**Published:** 2018-06-01

**Authors:** Nuwan Darshana Wickramasinghe, Devani Sakunthala Dissanayake, Gihan Sajiwa Abeywardena

**Affiliations:** 1 Department of Community Medicine, Faculty of Medicine and Allied Sciences, Rajarata University of Sri Lanka, Saliyapura, 50008 Sri Lanka; 2 Department of Community Medicine, Faculty of Medicine, University of Peradeniya, Peradeniya, 20400 Sri Lanka; 3 Teaching Hospital-Kandy, Kandy, 20000 Sri Lanka

**Keywords:** Student burnout, MBI-SS, Prevalence, Correlates, Collegiate cycle, Sri Lanka

## Abstract

**Background:**

Even though the concept of burnout has been widely explored across the globe, the evidence base on burnout among high school students in the South Asian context is scanty. Against the backdrop of ever-increasing educational demands and expectations, the present study was designed to determine the prevalence and correlates of burnout among collegiate cycle students in Sri Lanka.

**Methods:**

A school-based cross-sectional study was conducted among 872 grade thirteen students in 15 government schools in an educational zone, Kegalle district, Sri Lanka selected by a stratified cluster sampling technique. The validated Sinhala version of the 15-item Maslach Burnout Inventory-Student Survey (MBI-SS) was used to assess burnout. The adjusted prevalence of burnout was computed based on the clinically validated cut-off values using the “exhaustion + 1” criterion. Multivariable logistic regression was carried out using backward elimination method to quantify the association between burnout and selected correlates identified at bivariate analysis at p value less than 0.05.

**Results:**

The response rate was 91.3% (n = 796). The adjusted prevalence of burnout among grade thirteen students was 28.8% (95% CI = 25.0–32.7%). Multivariable analysis elicited a multitude of statistically significant associations with burnout when controlled for other factors included in the model (p < 0.05). Perceived satisfaction related to the school environment (classroom and library facilities), school curriculum (scope, relevance, and difficulty of the subject content), study enthusiasm (preferred subject stream), study support (support from parents to teachers), and future expectations (personal and parental expectations) emerged as statistically significant negative associations with burnout, whereas having to encounter disturbances while studying and being subjected to bullying at school emerged as statistically significant positive associations with burnout.

**Conclusions:**

The burnout prevalence among grade thirteen students in the selected educational zone, Sri Lanka is high. Most of the significant correlates of burnout are directly related to the academic endeavours. It is recommended to strengthen the counseling services at the school level to rectify the problems related to burnout among collegiate cycle students in Sri Lanka.

## Background

The concept of student burnout has been in the limelight since the introduction of the Maslach Burnout Inventory-Student Survey (MBI-SS) in 2002. In accordance with the original definition of burnout, student burnout is defined as, “a three-dimensional syndrome that is characterised by feelings of exhaustion due to the demands of studying, a cynical attitude of withdrawal and detachment, and reduced professional efficacy regarding academic requirements” [1]. In the global literature on student burnout research, the MBI-SS has been cited as the most widely used research instrument to assess burnout in different student populations [2, 3]. Furthermore, the validity and the reliability including the three-factor conceptualisation of MBI-SS have been confirmed in a number of different student populations in different countries [1, 3–6].

Amongst the wealth of research concerning student burnout, a vast majority has focused on assessing burnout among university undergraduates [1, 3, 7, 8]. However, against the backdrop of ever-increasing educational demands and expectations, the amount of research conducted among high school students is scanty. Moreover, within the scanty evidence base, the reported prevalence of burnout reflects a substantial variation depending on the definitions and criteria used, intrinsic factors of the samples studied, and the cut-off values applied for the assessment. The prevalence of burnout among Finnish junior high school students was reported as 10.9% [9], while other studies have reported even higher prevalence estimates among high school students [10, 11].

Studies conducted to explore the associations of burnout among different student populations across the globe have revealed a multitude of significant associations. Regarding high school and college students, characteristics such as lower self-efficacy [12, 13], maladaptive perfectionism [14], negative temperament [15], negative self-image [16], and depressive symptoms [17] are found to have positive associations with burnout. Educational environment related factors such as positive school class atmosphere [18], support from school [18], and academic achievement [12, 15, 17] are negatively associated, while high course/work load is positively associated with burnout [12, 15].

Even though the global literature suggests that there is a multitude of student burnout correlates, the evidence in the South Asian context is sparse. This existing research gap in the South Asian context needs to be addressed, as differences in the socio-cultural backgrounds, educational systems, and the level of education might limit the generalisability of previous study findings.

The scanty evidence base pertaining to student burnout in the South Asian context reveals that the prevalence of burnout among Indian undergraduate and postgraduate students substantially varies from 10.2 to 52.0% [19, 20]. While there is a paucity of literature assessing burnout among high school students in the South Asian context, thus far there is no published literature related to student burnout in Sri Lanka.

In Sri Lanka, the collegiate cycle in the education system (consists of grade 12 and grade 13) leads to the General Certificate of Examination Advanced Level, which is the national level selection examination for state university admissions. National statistics indicate that the examination has become extremely competitive for Sri Lankan collegiate cycle students, who are in the age range of 17–19 years [21]. Evidence suggests that approximately 40% of adolescents found it stressful to cope with the academic pressures exerted on them by parents and teachers [22] and almost one in five adolescents in schools have clinically relevant mental health problems with a substantial proportion having symptoms classified as definite or severe, while educational performance is reported as the most impacted area of life [23]. Furthermore, the prevalence of mental health problems such as depression and anxiety among the collegiate cycle students in Sri Lanka is high with examinations being the most commonly cited cause [24]. Though academic endeavours are usually considered as the ostensible reason for the resultant mental health problems, other numerous non-academic factors could have been the significant contributive reasons. In the light of high prevalence of mental health problems in students, it is of utmost importance to explore the concept of student burnout, which is directly assessing psychological well-being in relation to academic endeavours.

Given the research vacuum pertaining to student burnout in Sri Lanka, the present study was designed to assess the prevalence and correlates of burnout among collegiate cycle students in a selected educational zone in Sri Lanka.

## Methods

### Study design and setting

This school-based cross-sectional study was conducted in a selected educational zone in the Kegalle district, Sabaragamuwa Province, Sri Lanka from January 2015 to April 2015. Altogether there are 144 schools in the selected educational zone with a total student population of approximately 51,000.

### Participants

The study population consisted of all grade thirteen students studying in Sinhala medium government schools in the educational zone at the time of the study excluding students who were unable to read or write in Sinhala language. There were 1690 grade thirteen students in seven category 1AB and 31 category 1C government schools studying in four main subject streams, viz., Science, Arts, Commerce and Technology. Category 1AB schools have classes in all subject streams, whereas 1C schools have classes in Arts and Commerce streams only.

The required sample size was calculated with 95% confidence level, 5% absolute precision for an anticipated prevalence of burnout of 30% (as 32% of collegiate cycle students were categorised as having burnout in the validation study of the Sinhala version of the MBI-SS) based on standard sample size calculation formula [25] using OpenEpi software version 3.01. The final sample size was computed as 790, after adjusting for a design effect of 2.2 and an anticipated level of non-response of 10%. Stratified cluster sampling technique was used to select the study sample in which a cluster was defined as a grade thirteen class with a median number of 21 students. Hence, the study was conducted in 38 clusters from 15 schools selected according to the probability-proportional-to-size.

### Measures

A pre-tested, validated self-administered questionnaire (SAQ), which consisted of two components, was used in the present study.

The first component of the SAQ included the Sinhala version of the 15-item MBI-SS in order to assess the burnout status. The MBI-SS has been used to assess burnout status among high school students in several settings [3, 26–28] and the Sinhala version of the 15-item MBI-SS was found to be a valid and a reliable instrument to assess burnout among collegiate cycle students in Sri Lanka [29]. Out of the total 15 items of the MBI-SS, five, four and six items are targeted at identifying exhaustion (EX), cynicism (CY) and reduced professional efficacy (rPE) subscales, respectively. The frequency in which the respondents experience feelings related to each subscale was assessed using a seven-point, fully anchored response format, ranging from 0 (never) to 6 (every day).

During the validation and cultural adaptation of the Sinhala version of the MBI-SS, a multi-disciplinary panel of experts in the fields of psychiatry, psychology, public health, teaching, student counseling, and medical education has assessed each item of the questionnaire on its relevance, appropriateness, and acceptability in the local context for assessing burnout among grade thirteen students. In the confirmatory factor analysis, the 15-item three-factor model emerged as an acceptable fitting model. In addition, the 15-item MBI-SS showed high internal consistency (Cronbach’s α > 0.8) and high test–retest reliability (p < 0.001) [29].

The clinically validated cut-off values for the subscale scores of the MBI-SS were developed by computing Receiver Operating Characteristic curves using the clinical diagnosis made by a Consultant Psychiatrist as the reference standard in a sample of grade thirteen students in a similar educational setting. The clinical assessment of burnout by the Consultant Psychiatrist was based on the clinical criteria for the diagnosis of work related neurasthenia according to the ICD 10 classification [30–35]. Diagnostic accuracy of the MBI-SS test results based on “exhaustion + 1” criterion, which is an accepted criterion used in burnout research [30, 34–37], was assessed comparing with the results of the clinical diagnosis. The clinically validated cut-off values for EX, CY and rPE subscale scores were 12.5, 7.5 and 10.5, respectively. The sensitivity, specificity, positive and negative predictive values of the Sinhala version of the 15-item MBI-SS were 91.9, 93.2, 86.4 and 96.1%, respectively. Further, the positive and negative likelihood ratios were 13.48 and 0.09, respectively [38].

The second component of the SAQ, which was intended to gather information related to the correlates of burnout, was developed following an extensive literature search and with the inputs from the experts in the fields of psychiatry, psychology, public health, teaching, student counseling, and medical education. The questionnaire was pre-tested in a sample of grade thirteen students in a similar educational setting. The questionnaire consisted of ten sections focused on information related to personal and family characteristics, residence, school environment, curriculum, the pattern of study, support for studies, study enthusiasm, future expectations, personal behaviours/personal life factors, and behaviours of others.

### Procedure

Ethical clearance to conduct the study was obtained from the Ethics Review Committee of the Faculty of Medicine and Allied Sciences, Rajarata University of Sri Lanka (Reference no: ERC/2014/057). Prior to data collection, administrative clearance was obtained from the Zonal Director of Education and the principals of all the selected schools. The dates for data collection in different schools were selected according to the logistic convenience of the schools in order to minimise the disturbance to the routine academic and other endeavours. Informed written consent was obtained from all the students in each selected classroom. Data collection was took place inside the classrooms and each participant was given a copy of the printed SAQ to be filled independently. Confidentiality of data collected was adhered to strictly and the anonymity of the participants was maintained.

### Data analysis

Data analysis was done by using the SPSS version 17.0. After entering, double independent check of entries was carried out to identify any incompatible entries. The dataset was examined for univariate and multivariate outliers using box plots and Mahalanobis distance, respectively.

Scoring of the MBI-SS was carried out according to the MBI manual instructions [39]. Based on the clinically validated cut-off values for the three subscales, a participant was categorised as having burnout according to the “exhaustion + 1” criterion, in which a participant who is having a high score on EX in combination with a high score on either of the CY or rPE subscale was regarded as having burnout [30, 37]. As cluster sampling technique was used to select the study participants and the number of participants in the different clusters was not uniform, weighted analysis was conducted to determine the prevalence of burnout. Each observation in the sample was assigned a particular weight, which was calculated as the product of inverse selection probabilities at each stage of sampling [40, 41]. Incorporating the values of diagnostic accuracy of validated Sinhala version of the MBI-SS [38], the adjusted prevalence of burnout with 95% confidence interval (CI) was calculated in grade thirteen students [42].

For the assessment of correlates of burnout, a two-step procedure was followed. First, a bivariate analysis was performed to identify potential correlates of burnout. Then, a multivariable analysis using binary logistic regression was conducted to identify the relevant predictors of burnout and to control for potential confounding among the various predictor variables. Categorical data related to predictor variables were amalgamated rationally as dichotomous variables where necessary for the bivariate analysis and the crude odds ratios (OR) were calculated as the measures of effect with 95% CI. Correlates that showed statistical significance at p value less than 0.05 in the bivariate analysis were included in the multivariable analysis using backward stepwise elimination method. The model produced adjusted odds ratios (AOR) and 95% CI with the significance level for variables of interest. In order to conduct logistic regression, it is recommended to have at least ten observations per an independent variable [43] and the dataset met this criterion.

## Results

### Characteristics of the sample

Out of the total of 872 grade thirteen students in the 38 identified clusters, 796 students completed the SAQ; hence, the response rate was 91.3%. Table 1 summarises the basic characteristics of the study sample.

**Table 1:** Basic characteristics of the sample of grade thirteen students (n = 796)

Characteristic	Number	Percentage (%)	Cumulative percentage (%)
Sex			
Female	440	55.3	55.3
Male	356	44.7	100.0
Religion			
Buddhist	774	97.2	97.2
Hindu	2	0.3	97.5
Christian	16	2.0	99.5
Islam	1	0.1	99.6
Other	3	0.4	100.0
Monthly family income			
LKR 10,000–20,000	251	31.5	31.5
LKR 20,001–30,000	118	14.8	46.4
LKR 30,001–40,000	98	12.3	58.7
LKR 40,001–50,000	143	18.0	76.6
> LKR 50,001	186	23.4	100.0
Subject stream			
Science	235	29.5	29.5
Arts	276	34.7	64.2
Commerce	229	28.8	93.0
Technology	56	7.0	100.0
Total	796	100.0	

*LKR* Sri Lankan Rupees

The mean age of the grade thirteen students in the sample was 18.4 years (SD = 0.32 years). The majority of the participants were females (n = 440, 55.3%) and 276 students (34.7%) were studying in the Arts subject stream.

### Descriptive statistics of the Sinhala version of the MBI-SS subscale scores

Table 2 summarizes the mean total scores and the mean item scores of the three subscales of the MBI-SS Sinhala version.

**Table 2:** Descriptive statistics of the subscale scores of the MBI-SS Sinhala version among grade thirteen students (n = 796)

Subscale	Mean total score	SD	Mean item score	SD
EX	11.98	7.16	2.40	1.43
CY	6.80	5.98	1.70	1.49
rPE	10.56	6.46	1.76	1.08

### Prevalence of burnout

The prevalence of burnout based on the clinically validated cut-off values for each subscale score and the “exhaustion + 1” criterion was 36.8% (95% CI = 33.5–40.2%). The weighted analysis conducted to compensate for the complex sampling design resulted in a weighted prevalence estimate of 31.3% (95% CI = 28.1–34.6%). According to the sensitivity and the specificity of the Sinhala version of the 15-item MBI-SS, the adjusted prevalence of burnout among grade thirteen students in the study was 28.8% (95% CI = 25.0–32.7%).

### Correlates of burnout in the bivariate analysis

In the bivariate analysis, 35 factors emerged as significant predictors of burnout. These included a number of factors related to the study environment, curriculum, and behaviours. Table 3 presents the summary of statistically significant independent predictor variables of burnout emerged in the bivariate analysis.

**Table 3:** Statistically significant independent predictor variables of burnout in grade thirteen students in the bivariate analysis

Characteristic	Burnout	No burnout	Total	Odds ratio (95% CI) p value
	n (%)	n (%)	n (%)	
Personal characteristics				
Sex (n = 796)				
Female	145 (33.0)	295 (67.0)	440 (100.0)	0.7 (0.5–0.9)
Male	148 (41.6)	208 (58.4)	356 (100.0)	p = 0.012
Residence related factors				
Satisfaction of study place (n = 705)^a^				
Satisfactory	139 (32.0)	295 (68.0)	434 (100.0)	0.6 (0.5–0.9)
Not satisfactory	116 (42.8)	155 (57.2)	271 (100.0)	p = 0.004
Type of disturbances (n = 360)^a^				
From inside	70 (49.3)	72 (50.7)	142 (100.0)	1.5 (1.0–2.3)
From outside/both inside and outside	85 (39.0)	133 (61.0)	218 (100.0)	p = 0.054
Disturbances to studies at residence (n = 796)				
Yes	155 (43.1)	205 (56.9)	360 (100.0)	1.6 (1.2–2.2)
No	138 (31.7)	298 (68.3)	436 (100.0)	p = 0.001
Duration of travel to school (n = 796)				
Less than 30 min	131 (33.0)	266 (67.0)	397 (100.0)	0.7 (0.5–0.9)
More than 30 min	162 (40.6)	237 (59.4)	399 (100.0)	p = 0.026
School environment related factors				
Facilities of the classroom (n = 796)				
Satisfactory	81 (17.0)	395 (83.0)	476 (100.0)	0.1 (0.1–0.2)
Not satisfactory	212 (66.3)	108 (33.7)	320 (100.0)	p < 0.001
School library facilities (n = 796)				
Satisfactory	194 (33.1)	392 (66.9)	586 (100.0)	0.6 (0.4–0.8)
Not satisfactory	99 (47.1)	111 (52.9)	210 (100.0)	p < 0.001
School health services (n = 796)				
Satisfactory	111 (30.4)	254 (69.6)	365 (100.0)	0.6 (0.4–0.8)
Not satisfactory	182 (42.2)	249 (57.8)	431 (100.0)	p = 0.001
School counseling services (n = 796)				
Satisfactory	98 (30.9)	219 (69.1)	317 (100.0)	0.7 (0.5–0.9)
Not satisfactory	195 (40.7)	284 (59.3)	479 (100.0)	p = 0.005
School recreational facilities (n = 796)				
Satisfactory	85 (28.3)	215 (71.7)	300 (100.0)	0.5 (0.4–0.7)
Not satisfactory	208 (41.9)	288 (58.1)	496 (100.0)	p < 0.001
Curriculum related factors				
Scope of subject content (n = 766)^a^				
Satisfactory	115 (22.5)	395 (77.5)	510 (100.0)	0.1 (0.1–0.2)
Not satisfactory	175 (68.4)	81 (31.6)	256 (100.0)	p < 0.001
Amount of assignments/workload (n = 777)^a^				
Satisfactory	103 (28.5)	259 (71.5)	362 (100.0)	0.5 (0.4–0.7)
Not satisfactory	180 (43.7)	232 (56.3)	412 (100.0)	p < 0.001
Relevance of subject area (n = 781)^a^				
Satisfactory	141 (30.5)	322 (69.5)	463 (100.0)	0.5 (0.4–0.7)
Not satisfactory	144 (45.3)	174 (54.7)	318 (100.0)	p < 0.001
Difficulty in understanding (n = 796)				
Easily understood	79 (18.9)	338 (81.1)	417 (100.0)	0.2 (0.1–0.3)
Not easily understood	214 (56.5)	165 (43.5)	379 (100.0)	p < 0.001
Study pattern related factors				
Duration of study per day (n = 796)				
Less than 2 h	119 (42.5)	161 (57.5)	280 (100.0)	1.4 (1.1–2.0)
More than 2 h	174 (33.7)	342 (66.3)	516 (100.0)	p = 0.014
Methods of study (n = 796)				
Interactive methods with others	271 (36.0)	482 (64.0)	753 (100.0)	0.5 (0.3–0.9)
No interaction with others	22 (51.2)	21 (48.8)	43 (100.0)	p = 0.045
Answering past papers (n = 796)				
Yes	177 (31.8)	379 (68.2)	556 (100.0)	0.5 (0.4–0.7)
No	116 (48.3)	124 (51.7)	240 (100.0)	p < 0.001
Study support related factors				
Support from parents (n = 792)				
Satisfactory	208 (30.4)	476 (69.6)	684 (100.0)	0.1 (0.1–0.2)
Not satisfactory	84 (77.8)	24 (22.2)	108 (100.0)	p < 0.001
Support from colleagues (n = 759)^a^				
Satisfactory	160 (33.8)	313 (66.2)	473 (100.0)	0.7 (0.5–1.0)
Not satisfactory	117 (40.9)	169 (59.1)	286 (100.0)	p = 0.050
Support from teachers (n = 780)^a^				
Satisfactory	231 (33.3)	462 (66.7)	693 (100.0)	0.3 (0.2–0.5)
Not satisfactory	54 (62.1)	33 (37.9)	87 (100.0)	p < 0.001
Study enthusiasm related factors				
Subject stream (n = 740)^a^				
Science	105 (44.7)	130 (55.3)	235 (100.0)	1.8 (1.3–2.5)
Arts/Commerce	154 (30.5)	351 (69.5)	505 (100.0)	p < 0.001
Preference of subject stream (n = 796)				
Own preference	230 (32.5)	478 (67.5)	708 (100.0)	0.2 (0.1–0. 3)
Not own preference	63 (71.6)	25 (28.4)	88 (100.0)	p < 0.001
Opinion of academic performance (n = 796)				
Satisfactory	112 (28.6)	279 (71.4)	391 (100.0)	0.5 (0.4–0.7)
Not satisfactory	121 (44.7)	224 (55.3)	405 (100.0)	p < 0.001
Average marks (n = 577)^a^				
Less than 50	101 (46.3)	117 (53.7)	218 (100.0)	1.6 (1.1–2.2)
More than 50	126 (35.1)	233 (64.9)	359 (100.0)	p = 0.007
Future expectation related factors				
Personal expectations (n = 774)^a^				
Encouraging	249 (34.8)	467 (65.2)	716 (100.0)	0.3 (0.2–0.6)
Not encouraging	36 (62.1)	22 (37.9)	58 (100.0)	p < 0.001
Expectations of parents (n = 772)^a^				
Encouraging	241 (33.8)	473 (66.2)	714 (100.0)	0.2 (0.1–0.3)
Not encouraging	44 (75.9)	14 (24.1)	58 (100.0)	p < 0.001
Expectations of teachers (n = 761)^a^				
Encouraging	232 (34.6)	439 (65.4)	671 (100.0)	0.4 (0.3–0.7)
Not encouraging	49 (54.4)	41 (45.6)	90 (100.0)	p < 0.001
Expectations of colleagues (n = 677)^a^				
Encouraging	162 (33.5)	322 (66.5)	484 (100.0)	0.7 (0.5–0.9)
Not encouraging	83 (43.0)	110 (57.0)	193 (100.0)	p = 0.020
Personal behaviours/personal life factors				
Love affair (n = 796)				
Yes	87 (43.3)	114 (56.7)	201 (100.0)	1.4 (1.1–2.0)
No	206 (34.6)	389 (65.4)	595 (100.0)	p = 0.028
Effect of hobbies (n = 769)^a^				
Useful to studies	67 (31.8)	144 (68.2)	211 (100.0)	0.7 (0.5–1.0)
Not useful to studies	219 (39.2)	339 (60.8)	558 (100.0)	p = 0.055
Effect of religious activities (n = 780)^a^				
Useful to studies	216 (35.1)	400 (64.9)	616 (100.0)	0.7 (0.4–1.1)
Not useful to studies	69 (42.1)	95 (57.9)	164 (100.0)	p = 0.098
Factors related to behaviours of other people				
Household substance abuse (n = 796)				
Yes	152 (46.2)	177.(53.8)	329 (100.0)	2.0 (1.5–2.7)
No	141 (30.2)	326 (69.8)	467 (100.0)	p < 0.001
Colleagues substance abuse (n = 796)				
Yes	130 (42.9)	173 (57.1)	303 (100.0)	1.5 (1.1–2.0)
No	163 (33.1)	330 (66.9)	493 (100.0)	p = 0.005
Peer pressure for substance abuse (n = 796)				
Yes	21 (53.8)	18 (46.2)	39 (100.0)	2.1 (1.1–3.9)
No	272 (35.9)	485 (64.1)	757 (100.0)	p = 0.024
Subjected to bullying at school (n = 796)				
Yes	160 (51.8)	149 (48.2)	309 (100.0)	2.9 (2.1–3.9)
No	133 (27.3)	354 (72.7)	487 (100.0)	p < 0.001

^a^Total is not equal to 796 due to responses not included in the analysis and/or missing values

### Multivariable analysis of correlates of burnout

All 35 independent predictors identified at bivariate analysis were included in the multivariable analysis. None of these predictors had categories with very few observations, both the dependent and the independent variables were dichotomous in nature, and there were no outliers in the data set. Table 4 summarises the results of the multivariable analysis of the correlates of burnout retained in the final model. Out of the 14 factors retained in the final model, 12 factors made unique statistically significant contributions at a p value less than 0.05.

**Table 4:** Correlates of burnout in the sample of 796 grade thirteen students in the multivariable analysis

Factor	B	SE	Wald	df	p value	Odds ratio	95% CI
Disturbance to studies: yes	0.740	0.366	4.083	1	*0.043*	*2.1*	*1.1–4.3*
Facilities of the classroom: satisfactory	− 2.151	0.327	43.156	1	*< 0.001*	*0.1*	*0.1–0.2*
Library facilities: satisfactory	− 0.691	0.332	4.338	1	*0.037*	*0.5*	*0.3–0.9*
School health services: satisfactory	− 0.578	0.328	3.080	1	0.079	0.6	0.3–1.1
Scope of subject content: satisfactory	− 1.464	0. 357	16.818	1	*< 0.001*	*0.2*	*0.1–0.5*
Relevance of subject area: satisfactory	− 1.900	0.421	20.333	1	*< 0.001*	*0.2*	*0.1–0.3*
Difficulty in understanding: easily understood	− 1.380	0.340	16.444	1	*< 0.001*	*0.3*	*0.1–0.5*
Duration of study: less than 2 h per day	0.574	0.325	3.127	1	0.077	1.8	0.9–3.4
Support from parents: satisfactory	− 1.800	0.488	13.612	1	*< 0.001*	*0.2*	*0.1–0.4*
Support from teachers: satisfactory	− 1.084	0.449	5.832	1	*0.016*	*0.3*	*0.1–0.8*
Preference of subject stream: own preference	− 1.995	0.486	16.825	1	*< 0.001*	*0.1*	*0.1–0.4*
Personal expectations: encouraging	− 1.251	0.576	4.716	1	*0.030*	*0.3*	*0.1–0.9*
Expectations of parents: encouraging	− 1.002	0.346	8.375	1	*0.004*	*0.4*	*0.2–0.7*
Subjected to bullying at school: yes	0.981	0.302	10.557	1	*0.001*	*2.7*	*1.5–4.8*

Italic values indicate significance of p value (p < 0.05)

*df* degree of freedom; *SE* standard error; *95% CI* 95% confidence interval

Multivariable analysis elicited several statistically significant associations with burnout when controlled for other factors included in the model (p < 0.05). Perceived satisfaction about the facilities available in the classroom, about the library facilities, and about the scope of the subject content covered in the curriculum showed statistically significant negative associations with burnout.

Students who thought the content covered in the subjects are relevant to the curriculum and students who easily understood the subject content taught in the curriculum had statistically significant lower likelihood of having burnout in comparison to their counterparts.

Satisfactory support from parents to satisfactory support from teachers were found to have statistically significant negative associations with burnout.

The students who selected the subject stream based on their own decision were less likely to have burnout as opposed to those who have selected the current subject stream for other reasons. Students who felt that both their own future expectations and parental expectations are encouraging their studies were less likely to have burnout as opposed to those who did not feel so.

Having to encounter disturbances while studying and being subjected to bullying at school emerged as statistically significant positive associations with burnout.

## Discussion

The present study, which was designed with the objective of determining the prevalence and correlates of burnout among grade thirteen students in Sri Lanka, addresses an important research vacuum in relation to burnout research in high school students in the South Asian context. The study findings reveal that almost one in four grade thirteen students is likely to have burnout and burnout is significantly associated with a multitude of academic environment related correlates.

Having a high response rate and computing adjusted prevalence by compensating for the sampling complexity and diagnostic uncertainty of the assessment tool provide valid and precise estimates for burnout among grade thirteen students in this study.

The prevalence of burnout among the collegiate cycle students in this study is higher than the reported values of prevalence of burnout among high school students conducted in different study settings, such as 10.9% among students in Finnish public junior high schools [9], 14% among Finnish high school students [10], and 12.6% among middle school and regular secondary school in Northern China [44]. According to the World Bank statistics, the total enrolment in tertiary education in Finland and Sweden are substantially higher than that of Sri Lanka; based on which, it can be argued that the competitiveness of the tertiary education enrolment examinations and academic endeavour related stress could be higher in the Sri Lankan context as opposed to the study settings in other highlighted countries, contributing to the observed difference of the magnitude of the problem of burnout. However, other possible explanations such as the crucial differences in the educational context, burnout assessment tool, assessment cut-off values, and assessment criteria have to be taken into consideration in critically evaluating the research findings for comparative purposes. Provided that there is no universally accepted diagnosis method of burnout, it is important to appreciate that the prevalence estimates reported in the present study is dependent upon the burnout assessment criteria used in the study.

The multivariable analysis revealed that the students who encountered disturbances while studying were more likely to have burnout in comparison to their counterparts. It can be assumed that the disturbances at study place causing distractions would make the students unable to study or concentrate on their studies. The resultant frustration may provoke a feeling of indifference or distant attitude towards work. The ultimate result would be a cynical attitude of withdrawal and detachment from academic activities, which is an important aspect of student burnout.

School environment related factors, such as having satisfactory classroom and library facilities was associated with lower likelihood of having burnout and these findings are consistent with the existing evidence [18]. It can be assumed that the limited facilities or resources available at school environment are unable to inspire students to work hard, though in a paradox, the limited resources necessitate students to work hard to achieve better results. The lack of psychological stimulation originated by the lack of resources may aggravate students’ disinterest towards studies. Furthermore, it may contribute to further mental exhaustion of students owing to the idea of having to work hard. These reasons may have lead to the significant association elicited in the study.

Curriculum related factors, such as students’ satisfaction of the scope of the subject content covered in the curriculum and relevance of the subject to the curriculum, emerged as statistically significant predictors with lower likelihood of having burnout. In a similar vein, studies suggest a positive association of course workload with burnout [12, 15]. Factors such as the wide scope covered in the subject content and the difficulty in understanding the subject content demand students to make an extra effort in relation to their academic endeavours. Students in failing to understand the deep and difficult underpinning subject content resort to cram facts and theories, which hinders the opportunity to acquire higher-order generic competencies. This results in the imbalance between the abilities of the students and the expectations demanded by the academic environment leading to the excess amount of stress. This progressive vicious phenomenon culminates in burnout, which is recognised as a result of failed attempts to cope with a variety of negative stress situations [45].

In relation to support received for studies from others, students who perceived that they receive satisfactory support from their parents and satisfactory support from their teachers were found to have statistically significant negative associations with burnout and these findings are consistent with few other study findings [12, 13, 15]. Social support is regarded as one of the important aspects in explaining the burnout phenomenon [46]. Social support of an individual includes people who are caring and valuing the individual and the people that can be relied upon [12]. Social support has been identified as a resource that enables individuals to cope with stress [47]. Pines and Maslach [48] highlighted the fact that the social support has been identified as both a preventive mechanism and a remedy against burnout. These collective evidence, elucidate the negative association elicited between burnout and the support from parents to teachers. When academic endeavour related stress increases, the students are in search for social support to cope with the stress and the mental pressure.

Students, who selected the subject stream that they are following based on their own decision, were less likely to have burnout as opposed to those who opted to follow the current subject stream for other reasons. Similarly, Vasalampi, Salmela-Aro and Nurmi [49] have shown that self-concordance was associated with student burnout. According to Ryan and Connell [50], even though it is believed that personal goals are self-determined, they are not solely originated based on the intrinsic values and personal interests. It is believed that individuals are inclined to adopt their goals for external reasons such as social pressure or expectations of others [51]. These arguments have generated the basis for the self-concordance model for goal selection [51, 52]. Furthermore, according to literature, pursuit of goals for internalised reasons promotes sustained efforts leading to a better goal progress [53].

In relation to future expectations on academic endeavours, students who felt that both their own future expectations and parental expectations are encouraging their studies were less likely to have burnout as opposed to those who felt these expectations are not encouraging to their studies. As discussed above, as per the self-concordance model for goal selection, a certain element of external influence is incorporated in the selection of academic goals. In the Sri Lankan context, parental expectations play an important role in this regard. Hence, the subjective perception of encouraging parental expectations together with personal expectations may bring about a better goal progress. Thus, the students who are having such subjective perceptions may be less likely to develop burnout.

In the present study, the students who were subjected to bullying at school were more likely to have burnout. This finding is congruent with existing evidence suggesting that bullying in schools has a negative impact on students’ mental health [54, 55]. Being bullied is a particularly intense and traumatic form of stress, which usually involves a loss of control, and poses a threat to a person’s wellbeing.

In sum, the present study revealed several significant correlates of burnout and the findings are congruent with other study findings. Interestingly, amongst the significant correlates, almost all were either correlates that are directly related to the academic endeavours or correlates that have a direct effect on academic endeavours.

### Limitations

There are some limitations related to the study that need to be taken into consideration in interpreting the results. Owing to the fact that the present study was conducted in a selected educational zone in the Kegalle district, Sri Lanka the generalisation of the study findings to other study settings should be done with caution, considering the educational and cultural differences. Due to the cross-sectional nature of the study, a temporal relationship between burnout and correlates could not be established based on the present study findings. In the present study, even though multivariable analysis accounted for confounding, the effect of unknown confounders could not be accounted for.

## Conclusions

The prevalence of burnout among grade thirteen students in the selected educational zone, Sri Lanka is high (28.8%, 95% CI = 25.0–32.7%). Multivariable analysis elicited multiple statistically significant correlates that are directly related to the academic endeavours, including a multitude of factors related to the school environment, school curriculum, study enthusiasm, study support, and future expectations. In light of these findings, it is recommended to encourage the positive and supportive involvement of parents and teachers for students’ academic endeavours and to strengthen the counseling services at the school level to rectify the problems related to burnout among collegiate cycle students in Sri Lanka. The present study findings broaden the scanty evidence base pertaining to the magnitude and the associations of student burnout in the South Asian context.

## Abbreviations

AOR: adjusted odds ratio
CY: cynicism
EX: exhaustion
LKR: Sri Lankan Rupees
MBI-SS: Maslach Burnout Inventory-Student Survey
OR: odds ratio
rPE: reduced professional efficacy
SAQ: self-administered questionnaire
SE: standard error
SD: standard deviation
95% CI: 95% confidence interval
